# DLPFC rTMS is more effective than sham or orbitofrontal stimulation for smoking cessation and alters frontal brain activity: A double-blind, sham-controlled randomized clinical trial

**DOI:** 10.1016/j.jpsychires.2026.02.053

**Published:** 2026-03-04

**Authors:** Xingbao Li, Kevin A. Caulfield, Matthew J. Carpenter, Viswanathan Ramakrishnan, Evan S. Herrmann, Morgan Dancy, Mark S. George

**Affiliations:** a Brain Stimulation Division, Department of Psychiatry, Medical University of South Carolina, Charleston, SC, 29425, United States; b Center for Biomedical Imaging, Medical University of South Carolina, Charleston, SC, 29425, United States; c Addiction Sciences Division, Department of Psychiatry, Medical University of South Carolina, Charleston, SC, 29425, United States; d Department of Public Health Science, Medical University of South Carolina, Charleston, SC, 29425, United States; e Division of Therapeutics and Medical Consequences, National Institute on Drug Abuse, NIH, North Bethesda, MD, 20852, United States; f Ralph H. Johnson VA Medical Center, Charleston, SC, 29425, United States

**Keywords:** Tobacco use disorder, Smoking cessation, Image-guided TMS, DLPFC, mOFC, Reward circuitry, Executive control circuitry

## Abstract

**Objective:**

Repetitive transcranial magnetic stimulation (rTMS) shows promise for smoking cessation, but its neural mechanisms remain unclear. It is unknown whether rTMS reduces smoking by enhancing executive control or suppressing reward-related brain activity. This study aimed to address this gap by comparing excitatory and inhibitory rTMS targeting distinct cortical circuits implicated in tobacco use disorder (TUD).

**Methods:**

In a double-blind, sham-controlled design, treatment-seeking individuals with TUD were randomized to 15 sessions of personalized, fMRI-guided, E-field–dosed rTMS: (1) sham rTMS, (2) excitatory 10 Hz rTMS over the left dorsolateral prefrontal cortex (L-DLPFC; 3000 pulses/session), or (3) inhibitory 1 Hz rTMS over the left medial orbitofrontal cortex (L-mOFC; 900 pulses/session). fMRI scans were conducted pre- and post- 15 sessions of rTMS. Primary outcomes were reductions in cigarettes per day (CPD) and changes in blood-oxygen-level–dependent (BOLD) activity.

**Results:**

Thirty-five of 46 participants completed the study (sham = 9; 10 Hz L-DLPFC = 12; 1 Hz L-mOFC = 14). The 10 Hz L-DLPFC group showed a significantly greater CPD reduction than 1 Hz L-mOFC and sham (−11.14 vs. −4.92 and −6.43, respectively; p < 0.0001, partial η^2^ = 0.135). rTMS to the L-DLPFC also increased prefrontal activity and decreased orbitofrontal activity (p < 0.05), with the degrees of CPD reduction correlating with enhanced activation in L-DLPFC (p < 0.05).

**Conclusions:**

Enhancing executive control via 10 Hz rTMS over the L-DLPFC was more effective for smoking reduction than suppressing reward circuitry with 1 Hz rTMS over the L-mOFC. Findings suggest that strengthening prefrontal regulation of reward processing is a key mechanism by which rTMS promotes smoking cessation.

**Trial registration:**

clinicaltrials.gov Identifier: NCT 04903028.

## 1. Introduction

Tobacco use remains the leading cause of preventable morbidity and mortality worldwide, and it is responsible for more than 8 million deaths annually (WHO, 2021). Tobacco Use Disorder (TUD) is a chronic, relapsing condition marked by strong nicotine dependence and persistent craving, with fewer than 10% of smokers achieving long-term abstinence despite repeated quit attempts (DHHS, 2020). Current first-line treatments, including seven FDA-approved pharmacotherapies—such as nicotine replacement, bupropion, and varenicline-offer only modest efficacy (Aubin et al., 2014; Ziedonis et al., 2017; Rigotti et al., 2022; Carpenter et al., 2023). High relapse rates are attributed to limited engagement, adverse effects, and heterogeneous neurobiological mechanisms underlying addiction (Anthenelli et al., 2016; Rigotti et al., 2022). These limitations underscore the need for novel, mechanism-based interventions that directly target neural circuits implicated in TUD.

Neuroimaging studies suggest that TUD is associated with dysregulation between executive control and reward networks. Patients with TUD have hypofunction in the dorsolateral prefrontal cortex (DLPFC) and hyperactivation in mesocorticolimbic regions, including the medial orbitofrontal cortex (mOFC) (Brody, 2006; Hayashi et al., 2013). This imbalance impairs self-regulation and enhances the salience of smoking cues. Restoring prefrontal control and suppressing maladaptive reward responses may therefore promote abstinence (Hanlon et al., 2013, 2018; Li et al., 2023). Repetitive transcranial magnetic stimulation (rTMS), a non-invasive neuromodulation technique, offers a promising means to achieve this by modulating cortical excitability in a frequency-dependent manner—high-frequency (HF) (≥5 Hz) stimulation enhances, while low-frequency (LF)(≤1 Hz) suppresses, neural activity (Chen et al., 1997; Wu et al., 2000). Thus, rTMS could be developed as a novel therapy for smoking cessation with its ability to enhance or suppress neural circuits (Petit et al., 2022; Li et al., 2023).

Meta-analyses and systematic reviews indicate that HF-rTMS to the prefrontal cortex reduces craving, decreases daily cigarette consumption, and improves short-term abstinence rates (Petit et al., 2022; Li et al., 2023; Tang et al., 2023; Mehta et al., 2024). Several randomized controlled trials (RCTs) targeting the DLPFC with HF-rTMS report beneficial effects on smoking-related outcomes (Li et al., 2023; Mehta et al., 2024). In contrast, evidence for LF-rTMS is less consistent, and few studies have directly compared HF vs LF stimulation (Rose et al., 2011). Moreover, positive findings are reported more frequently for DLPFC stimulation than for mOFC targets (Petit et al., 2022; Li et al., 2023; Mehta et al., 2024).

Despite promising results, prior studies are highly heterogeneous with respect to stimulation frequency, cortical target coil type, dosing approach, and number of sessions, all of which limit interpretability and reproducibility (Amiaz et al., 2009; Li et al., 2013b, 2020, 2022; Dinur-Klein et al., 2014; Zangen et al., 2021). Most trials rely on scalp-based targeting and motor threshold-based dosing, and few directly compare mechanistically distinct protocols across different cortical regions. Recent advances in functional MRI-guided targeting and electric field(E-field)-based dosing offer an opportunity to improve precision and clinical efficacy by personalizing stimulation parameters (Cash and Zalesky, 2024; Dannhauer et al., 2024).

The present study addresses these limitations by testing two mechanistically distinct, personalized rTMS interventions for TUD: excitatory 10 Hz rTMS over the DLPFC to enhance executive control, and inhibitory 1 Hz rTMS over the mOFC to suppress reward-related activity. Individualized cortical targets were identified using functional MRI, and the stimulation dose was optimized using E-field modeling. We hypothesized that (1) both active protocols would reduce cigarette consumption relative to sham, (2) 10 Hz DLPFC stimulation would increase executive control-related activity while 1 Hz mOFC stimulation would decrease reward network activity, and (3) neural changes would correlate with a reduction in smoking behavior. By directly comparing competing neural mechanisms using a personalized, circuit-based approach, this study advances the development of rTMS as a targeted intervention for smoking cessation.

## 2. Subjects and methods

### 2.1. Study design

Treatment-seeking smokers were randomized in a 1:1:1 ratio to receive: (1) sham rTMS (split evenly between sham 10 Hz over L-DLPFC and sham 1 Hz over L-mOFC); (2) active 10 Hz rTMS over L-DLPFC; or (3) active 1 Hz rTMS over L-mOFC. Randomization was performed and implemented by an independent statistician not involved in data collection. Six-digit treatment codes were stored in sealed opaque envelopes and revealed only after data analysis. Participants, TMS treatment technicians, and study investigators were all blinded to group assignments. Each group received 5 daily sessions per week for 3 consecutive weeks (15 total sessions). A follow-up visit occurred one month after the final session. fMRI scans were acquired at baseline and after the 15th session. The Medical University of South Carolina IRB approved the study, which was registered at clinicaltrials.gov (NCT04903028). Supplementary Methods #1 and eFig. 1 provide the study timeline and assessments; statistical power considerations are in Supplementary Methods #2.

### 2.2. Participants

The study was conducted at MUSC from May 2021 to July 2023. Potential participants were identified via flyers and internet advertisements. Individuals passing a telephone pre-screen completed in-person eligibility assessments and provided written informed consent. Eligible participants were adults aged 18–65 who smoked ≥10 cigarettes/day for at least one year, met DSM-5 criteria for TUD, were motivated to quit (“very likely” or “somewhat likely”), and had a negative drug screen. Exclusion criteria included current use of psychoactive substances other than nicotine or caffeine, contraindications to MRI or TMS, use of non-combustible tobacco products, or current smoking cessation medication use. Consecutive enrollment and standardized screening were employed to minimize selection bias and improve sample representativeness. As in any study that utilizes flyers and internet advertisements, there may have been unintentional selection bias toward individuals with higher functioning and higher socioeconomic status.

### 2.3. rTMS treatment

#### rTMS Parameters:

We utilized a MagVenture MagPro R30 with a double-blind capable Cool-B65 A/P coil (MagVenture, Alpharetta, GA, USA), equipped with a custom-built electric sham loop (Supplementary Methods #3 and e Fig. 2). rTMS parameters for each condition were as follows: (1) Sham group: Participants were evenly split between receiving sham 1 Hz rTMS over L-mOFC or sham 10 Hz rTMS over L-DLPFC. (2) Active 10 Hz rTMS group: Participants received 10 Hz rTMS with a pulse train duration (on time) of 5 s and an inter-train interval (off time) of 10 s (15-s cycle time) for 3000 pulses per session (15 min per treatment session). (3) Active 1 Hz rTMS group: Participants continuously received 1 Hz rTMS for 15 min with 900 pulses per session. Stimulation intensity was personalized via E-field modeling to standardize the amount of electromagnetic stimulation each participant received at the cortical level (Opitz et al., 2011; Thielscher et al., 2011; Caulfield et al., 2021a, 2021b). (Supplementary Methods #5).

Coil localization methods for L-DLPFC and L-mOFC are detailed in Supplementary Method #4.

### 2.4. Cue provocation

Previous studies have suggested that exposing participants to real-life smoking cues before high-frequency rTMS can reduce nicotine dependence(Li et al., 2013a,b; Dinur-Klein et al., 2014). We used a structured 1.5-min interaction with smoking paraphernalia (i.e., cigarettes, ashtray, lighter) before each session (Carpenter et al., 2014). Concurrent with rTMS stimulation, participants watched a 15-min smoking-cue video on an iPad placed at the foot of the treatment chair (Li et al., 2020). (Supplementary Methods #6) Participants were asked to refrain from smoking for at least 2 h before each visit.

### 2.5. Clinical outcomes

#### Primary Outcomes:

Self-reported smoking data were collected via cigarette diaries (Timeline Follow-Back assessment) (Sobell et al., 1988). Cigarettes per day (CPD) were tracked over the 19-day treatment (15 session days plus 4 weekend days). The primary outcome was a reduction in CPD from baseline. We chose reduction in CPD, a sensitive surrogate measure that can detect treatment effects in this preliminary study, rather than binary abstinence outcomes used in later, larger-scale clinical trials.

#### Secondary Outcomes:

Daily secondary outcomes included a smoking craving visual analog scale (VAS, 0-7) and exhaled breath Carbon Monoxide (CO) measured by a Micro Smokerlyzer. Weekly secondary measures included the Fagerstrom Test for Nicotine Dependence (FTND) (Heatherton et al., 1991), Minnesota Nicotine Withdrawal Symptoms (MNWS) (Hughes and Hatsukami, 1986), the Questionnaire on Smoking Urges (QSU)-brief (Cox et al., 2001), and urine cotinine.

### 2.6. Functional MRI

Smoking Cue Craving (Li et al., 2013a, 2017a,b, 2024; Li et al., 2017a,b) and Resisting Urge to smoke (Hartwell et al., 2011, 2013) fMRI scans were acquired before and after the 15-session (3-week) rTMS treatment using a Siemens 3T PRISMA scanner with a 32-channel head coil at MUSC. Task fMRI utilized a gradient echo-planar imaging sequence (TR = 2200 ms, TE = 35 ms, 3 × 3 × 3 mm voxels, 328 vol, 36 slices). During scanning, participants rated their urge to smoke (scale 1–5) in response to smoking images. Whole-brain and the region of interest (ROI) analyses with SPM12 (Friston et al., 1994) compared activity in the left DLPFC and the left mOFC before and after treatment. For each ROI, a 6 mm sphere was centered on the peak activation. Pearson correlations examined associations between primary outcomes and ROI beta values. We reported detailed image analysis methods in Supplementary Methods #8.

### 2.7. Statistical analysis

All analyses were conducted using IBM SPSS Statistics 29, with a two-tailed significance level set at *p* < 0.05. Baseline group differences were examined using one-way ANOVA or χ^2^ tests. Our primary hypothesis was that 10 Hz rTMS over the L-DLPFC would yield greater reductions in CPD than 1 Hz L-mOFC or sham. To analyze longitudinal outcomes (CPD, CO, VAS, FTND, MNWS, and urine cotinine), we utilized mixed models for repeated measures (MMRM) with treatment group, time, and their interaction as fixed effects and subject as a random effect. Post-hoc pairwise comparisons were FDR-corrected for multiple comparisons. Effect sizes were reported as Cohen's *d* for pairwise contrasts and partial η^2^ for omnibus effects.

For neuroimaging data, pre- and post-treatment fMRI BOLD responses were analyzed using a general linear model with cluster-level FDR correction (*p* < 0.05). Regions of interest included the left DLPFC and mOFC. Changes in BOLD activity were correlated with behavioral outcomes to assess whether rTMS modulated executive control and reward circuitry. Model assumptions were checked for normality and variance homogeneity, and Greenhouse–Geisser corrections were applied when appropriate. Missing data were handled under the missing-at-random assumption using likelihood-based estimation.

## 3. Results

### 3.1. Enrollment

**Fig. 1.**
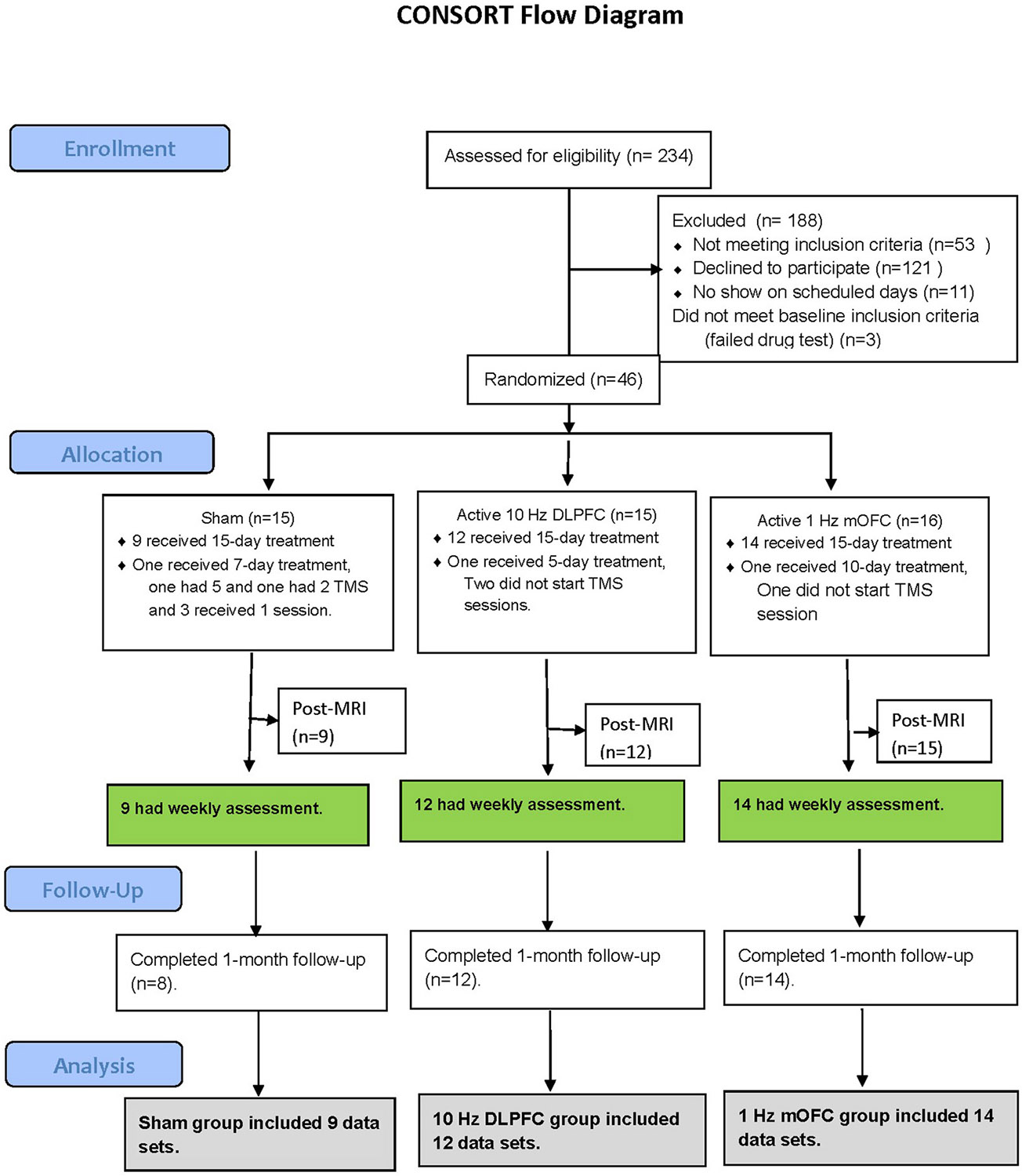
CONSORT diagram of participant flow The Flow of participants from a longitudinal cohort in the trial of active 10 Hz over the left DLPFC or active 1 Hz over the left mOFC or sham rTMS. The trial timeline includes the number of participants and dropouts in each phase. Of the 234 potential participants contacted, 111 were selected for a phone screen, and 46 were deemed eligible for the study. Of these 46 participants, 15 were assigned to receive Sham TMS (DLPFC or mOFC), 15 received active 10 Hz rTMS over the left DLPFC rTMS, and 16 received active 1 Hz rTMS over the mOFC. For analysis, completer cases were defined as randomized patients who received 15 sessions of rTMS treatment and underwent two MRI scans (baseline and post-rTMS). Overall, 35 study participants were included in the data analysis (sham = 9, 10 Hz DLPFC = 12, and 1 Hz mOFC = 14).

The progression through trial procedures is summarized in Consort Flow Diagram (Fig. 1).

### 3.2. Participant demographics and smoking characteristics

There were no significant differences between the three groups in baseline ratings, including age, gender, cigarette consumption, years of smoking, and FTND score (Table 1)

### 3.3. Clinical outcomes

#### Cigarettes Per Day (CPD):

**Fig. 2.**
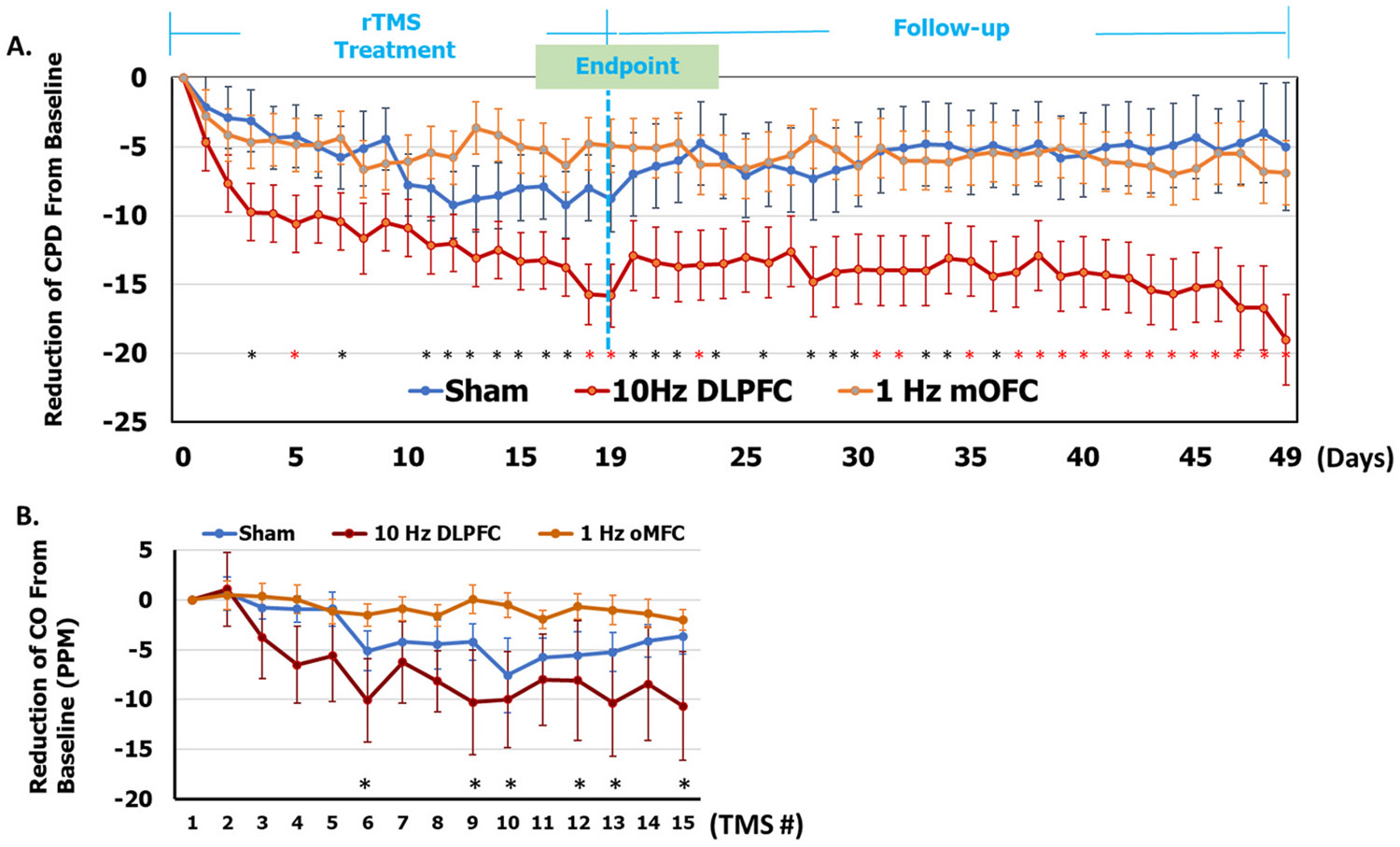
Reduction in cigarette consumption in the three treatment groups (Mean ± SE) (A) The primary endpoint was the reduction of CPD over 3 weeks of TMS treatment. The reduction of cigarettes per day (CPD) was defined as a reduced CPD from baseline (Screening visit). We collected CPD, as self-reported by the participants during rTMS treatment course (19 Days, including 15 treatments and weekends). For the 3-week TMS treatment course, the mixed model analysis reveals a significant treatment effect on the reduction of CPD (sham: −6.43[0.54]; 10 Hz DLPFC: −11.14[0.48]; 1 Hz mOFC: −4.92[0.43]; F_(2,623)_ = 48.65, p < 0.0001). Post-hoc tests showed that 10 Hz DLPFC group reduced significantly more CPD than did sham (p < 0.05) or 1 Hz mOFC (p < 0.001). In addition, the effect of 10 Hz DLPFC remained during the one-month follow-up period and even improved further. The results revealed a significant effect of treatment days (F_(29,623)_ = 2.23, p = 0.003). Overall, the 10 Hz DLPFC reduced more CPD than the sham and the 1 Hz mOFC (B) Consistent with CPD reductions above, mixed model analysis showed greater reductions in CO within DLPFC vs. sham and 1 Hz mOFC rTMS. The results showed a significant treatment effect on the reduction of CO level (sham: −3.45[0.86]; 10 Hz DLPFC: −7.00[0.75]; 1 Hz mOFC: 0.76[0.676]; F_(2,490)_ = 19.1, p < 0.0001; TMS session F_(14,493)_ = 1.54, p = 0.09). * In black color: The 10 Hz DLPFC rTMS group showed a more significant cigarette consumption reduction than the 1 Hz mOFC rTMS or sham treatment. * In red color: 10 Hz DLPFC treatment significantly reduced cigarette consumption more than both 1 Hz mOFC and sham treatments.

MMRM analysis revealed a significant treatment group effect on the reduction of CPD (sham: −6.43[0.54]; active 10 Hz L-DLPFC: −11.14[0.48]; active 1 Hz L-mOFC: −4.92[0.43]; and F_(2,623)_ = 48.65, p < 0.0001, partial η^2^ = 0.135) as well as treatment time effect (F_(18,623)_ = 1.96, p = 0.01, partial η^2^ = 0.054). Post-hoc tests showed that the 10 Hz L-DLPFC group had a more significant reduction in CPD than the sham condition (p < 0.05, Cohen's d = 2.87) and 1 Hz L-mOFC condition (p < 0.001, Cohen's d = 3.82) (Fig. 2A). This treatment effect persisted throughout the one-month follow-up period.

#### CO level:

**Fig. 3.**
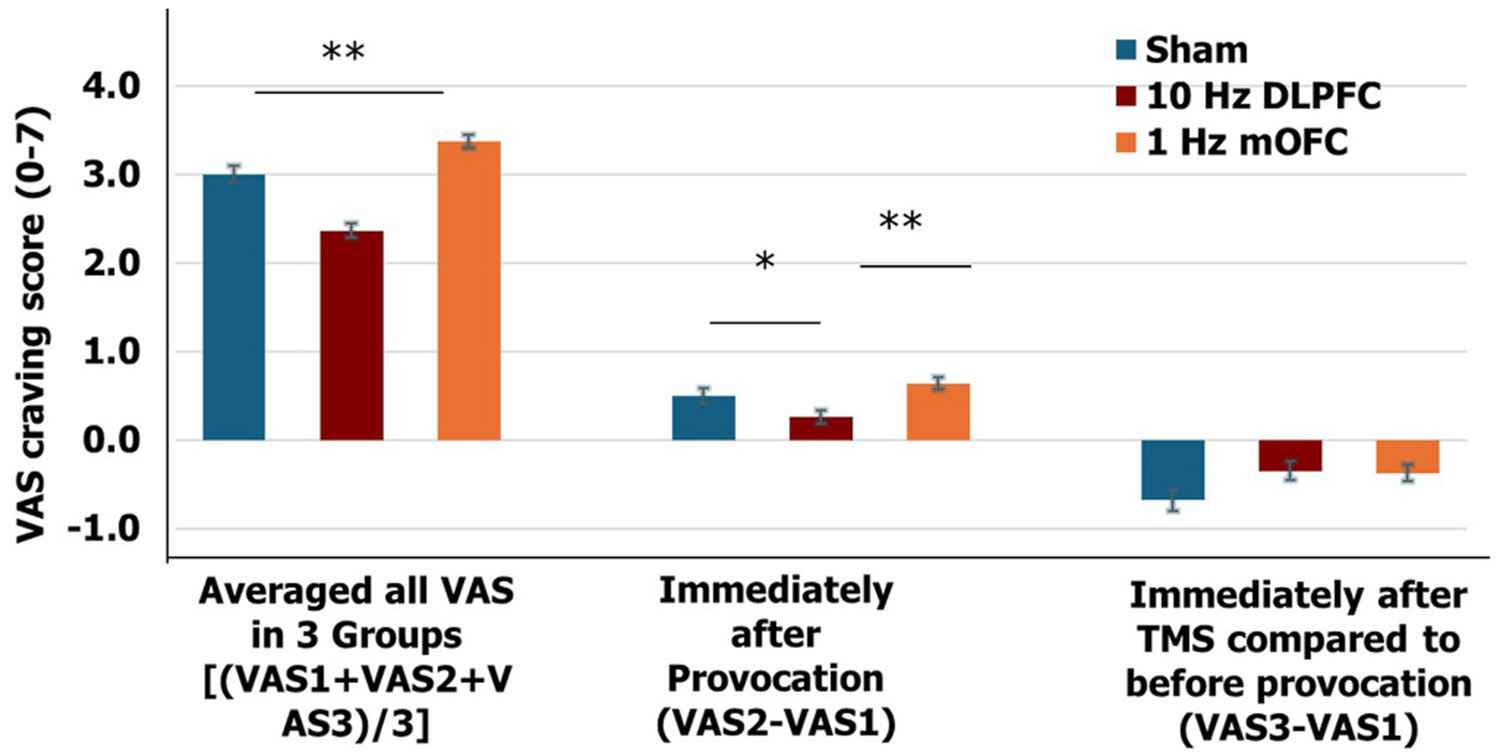
Effect of rTMS on acute cue craving in the visual analog scale in three treatment groups. (Mean ± SE) *Averaged all VAS in 3 Groups:* Pooled daily VAS craving ratings [(VAS 1 +VAS2 +VAS3)/3] for 15 sessions of rTMS, a mixed model revealed a significant treatment effect (F_(2,1485)_ = 42.61, p < 0.001). (Fig. 3. Left). *VAS2 minus VAS 1*: Using the acute change of VAS following provocation (VAS2 minus VAS1) as a dependent variable, a mixed model analysis (treatment group and rTMS treatment week (week 1, week 2, week 3), revealed a significant treatment effect (sham: 0.50[0.08]; 10 Hz DLPFC: 0.26[0.07]; 1 Hz mOFC: 0.64 [0.07]; F_(2,530)_ = 7.42, p < 0.001), no significant treatment week effect(_(2,530)_ = 2.69, p > 0.05). Post hoc analysis showed that 10 Hz DLPFC treatment had less acute provocation craving than either sham (p < 0.05) or mOFC (p < 0.01) (Fig. 3. Middle) *VAS3 minus VAS1:* Using the acute change of VAS following rTMS session (VAS3 minus VAS2) as a dependent variable, mixed model analysis (treatment group and rTMS treatment week (week 1, week 2, week 3) did not reveal any significant treatment group and treatment week effect (Fig. 3. Right) VAS: Visual analog scale for cue craving (1-7) VAS1: VAS rating before cue-provocation; VAS 2: VAS rating after cue-provocation and before rTMS session; VAS 3: VAS rating immediately after rTMS treatment session.

MMRM analysis revealed a significant treatment group effect (sham: 3.45[1.94]; 10 Hz DLPFC: −7.00[1.68]; 1 Hz mOFC: −0.75 [1.5]; F_(2,480)_ = 18.3, p < 0.0001). Post hoc analysis showed that 10 Hz L-DLPFC treatment reduced CO levels more than L-mOFC (p < 0.0001) and sham (p < 0.01). We did not find a significant main effect of time (F (14,480) = 1.49, p = 0.11; Fig. 2B). However, overall, the observed changes in breath CO levels were robustly consistent with changes in self-reported smoking behavior (CPD). See Supplementary Results #2 and e Fig. 3 for creatinine-adjusted cotinine.

#### Immediate Effect of rTMS on Craving (VAS):

Pooling three VAS scores in MMRM analysis, we found a significant treatment effect on the VAS (sham: 2.99[0.09]; 10 Hz L-DLPFC: 2.36[0.08]; 1 Hz L-mOFC: 3.37 [0.07]; F_(2,1485)_ = 42.61, p < 0.001), significant TMS number effect (F_(2,1485)_ = 8.81, p < 0.0001) and a significant provocation effect (F_(2,1485)_ = 31.85, p < 0.0001). Overall, the VAS score of the 10 Hz rTMS group was significantly less than 1 Hz rTMS group (p < 0.0001) and the sham group (p < 0.0001) (See Fig. 3 left panel). In the 10 Hz rTMS group, the provocation effect (VAS 2 minus VAS 1) was less than 1 Hz group (p < 0.01) and the sham group (p < 0.05) (See Fig. 3 middle panel).

#### Questionnaire of Smoking Urges-Brief (QSU-B):

MMRM analysis (3 [treatment conditions] x 5 measured timepoints [baseline, 6th TMS session, 10th TMS session, 15th TMS session, and 1-month follow-up]) revealed that 10 Hz L-DLPFC group had significantly lower mean craving ratings over 5 longitudinal measurements than did the sham and 1 Hz L-mOFC groups (sham: 27.34[15.65]; 10 Hz L-DLPFC: 22.07 [13.58]; 1 Hz L-mOFC: 29.25[14.97]; F_2,157_ = 4.49, p = 0.013), and that the craving rating continuously decreased during the TMS treatment course and remained at this level at follow-up visit (baseline: 36.08 [15.88], 6th TMS: 29.50[14.08], 10th TMS: 24.83[12.94], 15th TMS: 20.72[12.78], 1-month follow-up: 19.77[13.12]; F _4,157_ = 8.80, P < 0.001).

#### Smoking Abstinence Rate:

**Fig. 4.**
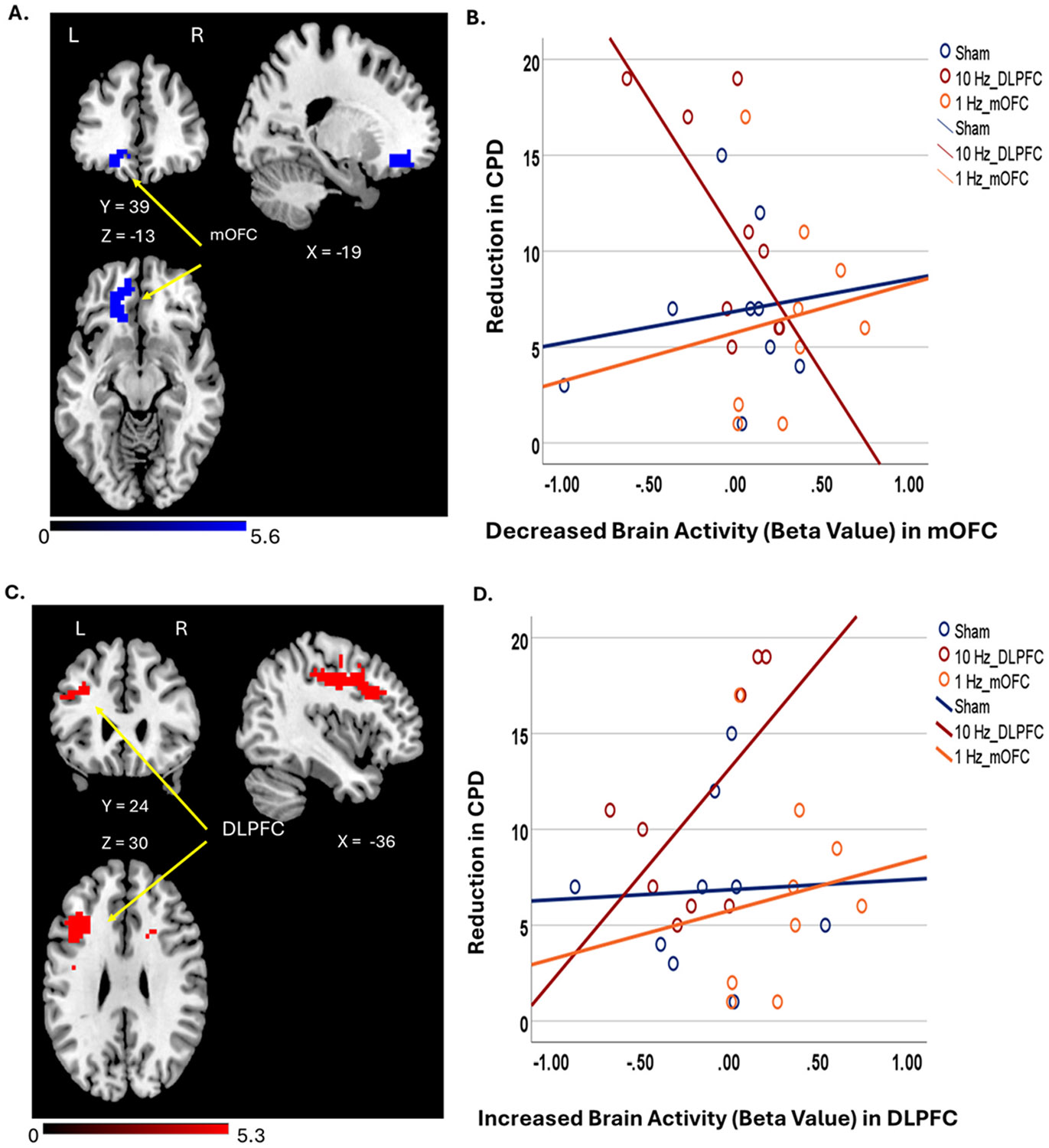
Clinical effect of rTMS associated with the change of brain activity (A). 10 Hz DLPFC rTMS reduced brain activity in mOFC, which is associated with a reduction in cigarette consumption during Cue Crave MRI scans. (0.05 FWE for cluster and voxel threshold p < 0.001). No significant treatment effects were observed in either the 1 Hz mOFC or sham group. (B). Decreased brain activity in mOFC (region of interest, 6 mm sphere) is likely associated with reduced CPD only after 10 Hz rTMS group in Cue Crave MRI scans (r = −0.81, p < 0.001). After removing the outlier, the correlation between reduced CPD and decreased brain activity became non-significant but tended to show a difference (r = −0.47, p = 0.14). Dark blue: Sham group (r = 0.15, p > 0.05), Dark red: 10 Hz DLPFC group; Orange: 1 Hz mOFC group (r = 0.00, p > 0.05) (C). 10 Hz rTMS increased brain activity in the DLPFC (p < 0.001, FWE cluster and voxel threshold). No significant treatment effects were observed in either the 1 Hz mOFC or sham group. (D). Increased brain activity in DLPFC (ROI, 6 mm sphere) is significantly associated with reduced CPD only after 10 Hz rTMS DLPFC group in Resisting Crave MRI scans (r = 0.89, p < 0.001). After removing the outlier, the correlation between reduced CPD and increased brain activity in the left DLPFC remained significant (r = 0.62, p < 0.05). Dark blue: Sham group (r = 0.00. p > 0.05), Dark red: 10 Hz DLPFC group; Orange: 1 Hz mOFC group (r = 0.17, p > 0.05) CPD: cigarettes per day FWE: Family-wise error.

This trial was not adequately powered to detect differences in smoking abstienence rate. There was no significant difference in the smoking abstinence rate between treatment groups at the end of rTMS course. Nonetheless, abstinence numerically favored the 10 Hz group (Supplementary Results #3 and e Fig. 4).

#### Safety and tolerability:

No serious adverse events occurred. Spontaneous side effects were recorded daily and categorized by common rTMS side effects (Supplementary Results #6and e Table 1). Pain was the most commonly reported side effect. In all cases, side effects subsided shortly after the stimulation session, and no participant required treatment.

### 3.4. Brain imaging outcomes

#### 3.4.1. Whole brain analysis results

##### Cue Craving Scans:

Paired *t*-test results comparing within-subject pre- to post-rTMS effects showed that 10 Hz TMS over L-DLPFC significantly decreased brain activity in L-mOFC (0.05 family-wise error [FWE] corrected for cluster and voxel threshold p < 0.001) (Fig. 4a). Neither the sham rTMS nor 1 Hz rTMS over L-mOFC significantly influenced brain activity from pre-to post-stimulation. (eFig. 5 and eTable 3 in Supplementary Results #7).

##### Resisting the Urge to Smoke Scans:

Paired *t*-test results showed that 10 Hz L-DLPFC rTMS increased brain activity in L-DLPFC, precentral gyrus, and dorsal ACC (0.05 FWE corrected for cluster and voxel threshold p < 0.001) (Fig. 4c) in the Resisting urge to smoke fMRI scan. We did not find any significant change after sham TMS or 1 Hz TMS over L-mOFC (eFig. 6 and eTable 3 in Supplementary Results #7).

##### Factorial Design Model Analysis:

For craving scans, three rTMS groups (sham, 10 Hz L-DLPFC, and 1 Hz L-mOFC) and two visit times (before and after rTMS treatment) were included in the factorial model. No interaction between brain activity and group or treatment time was found (p < 0.05 FWE for clusters and voxels, p < 0.001).

For resisting urge to smoke scans, the factorial model included three rTMS groups (sham, 10 Hz L-DLPFC, and 1 Hz L-mOFC) and two visit times (before and after rTMS treatment). No interaction of brain activity was found between groups and treatment time (p < 0.05 FWE for cluster and voxel threshold, p < 0.001).

#### 3.4.2. Region of interest (ROI) analyses

##### ROI analyses on cue-cravings fMRI:

The beta value from a 6 mm spherical area was extracted within the ROIs with the center of the local maximum z value for each ROI (−24, 39, −12). Paired *t*-test showed that 10 Hz rTMS reduced brain activity in the mOFC (Pre-rTMS: 0.77 [0.21]; Post-rTMS: 0.30 [0.10], t = 2.34, p < 0.05). Neither the sham rTMS (Pre-rTMS: 0.09[0.08]; Post-rTMS: 0.16[0.06], t = −0.64, p = 0.53) nor 1 Hz rTMS over L-mOFC(Pre-rTMS: 0.09[0.05]; Post-fMRI: 0.003[0.00], t = 1.69, p = 0.11) significantly influenced brain activity from pre-to post-stimulation.

##### ROI analyses on resisting urge to smoke fMRI:

The beta value from a 6 mm spherical area was extracted within the ROIs with the center of the local maximum z value for each ROI (group average MNI coordinate: −33, 21, 33). Paired *t*-test results showed a significant increase in DLPFC activity following rTMS (Pre-rTMS: −0.23 [0.15]; Post-rTMS: 0.26 [0.13], t = −5.33, p < 0.001). No significant changes were found for sham rTMS (Pre-rTMS: 0.03[0.11]; Post-rTMS: 0.05[0.06], t = −0.64, p = −0.22), or for 1 Hz rTMS over L-mOFC (Pre-rTMS: −0.07[0.04]; Post-fMRI: −0.01[0.00], t = −1.34, p = 0.20).

#### 3.4.3. Primary outcomes correlated with brain activity

The beta value from a 6 mm spherical area was extracted within the ROIs with the center of the local maximum z value for each ROI. Reductions in brain activity were likely associated with reduced CPD. The eigenvariates of the region of interest (6 mm sphere) were negatively correlated with the reduction in CPD from baseline (r = −0.81, p < 0.001). We identified one outlier in brain activity changes following 3 weeks of 10 Hz rTMS. After removing the outlier, the correlation between the reduction in CPD and decreased brain activity was not significantly different but was in the hypothesized direction (r = −0.47, p = 0.14) (Fig. 4b). We did not find a significant correlation between the reduction in CPD and decreased brain activity in mOFC after sham and 1 Hz rTMS treatment.

In addition, increased DLPFC activity was significantly associated with a reduction in CPD in the 10 Hz L-DLPFC group. The eigenvariate of the ROI (6 mm sphere) positively correlated with the reduction of CPD from baseline (r = 0.89, p < 0.001). After removing the outlier, the correlation between reduction in CPD and increased brain activity in L-DLPFC remained significant (r = 0.62, p < 0.05) (Fig. 4d). In contrast, we did not find a significant correlation between the reduction of CPD and increased brain activity in the DLPFC after sham and 1 Hz rTMS treatment.

### 3.5. Blind integrity

This was a well-blinded study at the time of treatment initiation. By the end of the study, participants correctly identified the sham condition less often than either of the active treatment groups (p = 0.03). Approximately 80% of participants in both the 10 Hz DLPFC and 1 Hz mOFC groups correctly identified their treatment, which was notably higher than in the sham group. However, confidence ratings did not differ significantly between treatment groups. Given the study design, which included one sham group (with an expected correct-guess rate of 33%) and two active groups (with an expected correct-guess rate of 67%), we believe the blinding was overall successful (p = 0.89). Additionally, the accuracy of operators' guesses did not differ significantly across groups (Supplementary Results #8 and e Table 3).

## 4. Discussion

This study provides the first direct comparison of excitatory and inhibitory rTMS protocols applied to distinct cortical circuits implicated in tobacco use disorder, using individualized fMRI-guided targeting and E-field–based dosing. We demonstrate that high-frequency (10Hz) stimulation for 15 sessions over three weeks of the left DLPFC produces meaningful reductions in daily cigarette consumption and craving relative to both low-frequency (1 Hz) stimulation of the left mOFC and sham rTMS. These behavioral improvements were accompanied by increased DLPFC activation and decreased mOFC activity, with reductions in cigarette use correlating with enhanced prefrontal engagement. Together, these findings support a circuit-level model in which strengthening executive-control networks yields downstream regulation of reward-related processes that sustain nicotine use, consistent with neurobiological models of addiction emphasizing prefrontal hypofunction and mesocorticolimbic hyperactivity (Goldstein and Volkow, 2011; Volkow and Morales, 2015).

Notably, the clinical effects of 10 Hz DLPFC stimulation were observed despite participants in this group having numerically higher baseline cigarette consumption and an earlier age of smoking initiation compared with the sham group—differences that were not statistically significant but may be clinically meaningful and are typically associated with greater nicotine dependence and poorer treatment outcomes (Breslau et al., 1993; Martinez-Ortega et al., 2008). When change in smoking was considered in proportional terms, the DLPFC group demonstrated the greatest reduction in cigarette consumption (approximately 50%), compared with the sham (36%) and mOFC (32%) groups, aligning with the primary finding that excitatory DLPFC stimulation produced the most robust clinical signal. The efficacy of DLPFC stimulation under these conditions suggests that augmenting executive-control circuitry may partially mitigate vulnerabilities associated with earlier initiation and more entrenched smoking behavior. Although the study was not powered to detect group differences in abstinence, the numerically higher quit rates observed in the 10 Hz DLPFC group further support its potential clinical relevance and warrant confirmation in larger, adequately powered trials that are underway.

From a mechanistic perspective, these findings align with prior evidence that rTMS modulates neural circuits supporting craving, cue reactivity, reward valuation, and inhibitory control (Hester and Garavan, 2004; Feil et al., 2010; Harmelech et al., 2023; Li et al., 2023). The DLPFC plays a central role in self-regulation and sop-down control, and high-frequency stimulation is known to increase cortical excitability and strengthen functional connectivity within frontostriatal and frontolimbic networks (Li et al., 2017, 2024, 2025). The observed pattern of increased DLPFC activation accompanied by reduced mOFC activity is consistent with this framework, suggesting that enhancing executive-control capacity indirectly constrains maladaptive reward processing. The correlation between rTMS-induced neural changes and reductions in cigarette use further supports the functional relevance of these circuit-level effects.

In contrast, low-frequency stimulation of the mOFC did not reduce cigarette use or craving and produced no measurable neuroimaging changes. Although inhibitory rTMS is often conceptualized as suppressing pathological hyperactivity, the mOFC serves a more integrative role in valuation, outcome monitoring, and adaptive decision-making (Goldstein and Volkow, 2011; Jenni et al., 2022). Global suppression of this region may therefore blunt both maladaptive and compensatory processes, limiting therapeutic efficacy. Methodological factors, including differences in stimulation frequency and pulse number between protocols, may also have contributed to the null effects observed. Notably, the limited efficacy of mOFC inhibition parallels recent findings from intermittent theta-burst stimulation studies targeting the same region, which similarly failed to reduce cigarette use despite measurable physiological effects (Mikellides et al., 2023; Addicott et al., 2024).

Our results extend a robust literature demonstrating that excitatory stimulation of executive-control regions is most consistently associated with reductions in smoking behavior. Multiple clinical trials have reported meaningful decreases in cigarette consumption and craving following high-frequency DLPFC stimulation across diverse samples and stimulation paradigms (Amiaz et al., 2009; Li et al., 2013, 2020, 2022; Dinur-Klein et al., 2014; Sheffer et al., 2018; Zangen et al., 2021). Neuroimaging studies further show that DLPFC stimulation modulates distributed control and reward networks, including reductions in cue-elicited activation with mesocorticolimbic regions such as the mOFC (Li et al., 2017, 2024). The current study advances this work by directly contrasting executive-control and reward-targeted interventions within a single experimental framework and by demonstrating convergent behavioral and neural effects that favor prefrontal excitation as a primary therapeutic mechanism.

Several limitations should be acknowledged. The modest sample size limited statistical power, particularly for secondary and neuroimaging outcomes, increasing the likelihood of Type II error. Replication in larger samples, focusing on more clinically meaningful outcomes (sustained, biologically verified abstinence) will be necessary to confirm these effects. Additionally, differences in stimulation parameters across protocols, including total pulse number, constrain frequency-specific interpretations and raise the possibility that pulse dose may have contributed to observed effects. Prior work has demonstrated that rTMS outcomes may vary as a function of cumulative pulse exposure and session dosing (Brunoni et al., 2017; Hsu et al., 2024), underscoring the need for studies that systematically equate and manipulate pulse dose within and across frequencies to clarify dos-response relationships. In the present study, pulse numbers were selected to align with established, mechanistically grounded protocols-excitatory high-frequency stimulation of the DLPFC and inhibitory low-frequency stimulation of the mOFC-rather than to equate total pulse dose across conditions. Finally, while personalized targeting may enhance mechanistic specificity, its clinical feasibility will depend on demonstrating clear advantages over simpler, more scalable approaches.

In summary, this study provides convergent behavioral and neuroimaging evidence that excitatory stimulation of the DLPFC is a more effective neuromodulatory strategy for reducing cigarette use and craving than inhibitory stimulation of the mOFC. By demonstrating that strengthening executive-control networks yields downstream regulation of reward circuitry, these findings refine mechanistic models of rTMS in tobacco use disorder and inform the rational design of future clinical interventions.

## Supplementary Material

Supplementary Material

## Figures and Tables

**Table 1:** Sample characteristics and group comparison results.

Characteristic	Sham (9)	DLPFC (12)	mOFC (14)	P value
Age	46.7(12.3)	55.1 (8.9)	52.6 (9.5)	Ns
Gender (M/F)	6/3	5/7	6/8	Ns
Years smoked	26.4(9.6)	37.0 (11.8)	33.9(11.9)	Ns
Age started smoking	19.2(4.9)	15.4 (5.3)	17.0(3.8)	Ns
Cigarette per day	17.6(6.6)	22.3 (11.4)	15.4 (5.2)	Ns
FTND score	5.2(2.2)	5.3 (1.8)	5.0(2.1)	Ns
Desire to quit smoking^a^	9.1(1.1)	9.3 (1.0)	9.2(1.2)	Ns
No. of prior quit attempts	2.8(1.4)	3.6(1.6)	3.5(1.3)	Ns

Mean (SD) is used for all continuous variables.

a On a scale of 0 to 10, with 10 representing the greatest desire to quit.

## Appendix A. Supplementary data

Supplementary data to this article can be found online at https://doi.org/10.1016/j.jpsychires.2026.02.053.
