# Supplementary material for: DLPFC rTMS is more effective than sham or orbitofrontal stimulation for smoking cessation and alters frontal brain activity: A double-blind, sham-controlled randomized clinical trial
Source: J Psychiatr Res. Author manuscript; Available in PMC 2026 Mar 27. (PMC13020188; doi:10.1016/j.jpsychires.2026.02.053)
Supplement: Supplementary Material [file NIHMS2155528-supplement-Supplementary_Material.docx]

**DLPFC rTMS is more effective than sham or orbitofrontal stimulation for smoking cessation and alters frontal brain activity: A sham-controlled randomized clinical trial**

**Supplemental Content**

**METHODS**

1. **Study overview**

**e Figure 1.** Timeline and assessments.


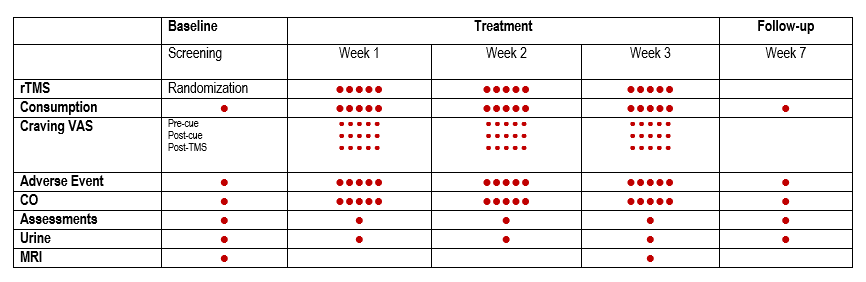


**e Figure 1. Timeline and assessments**. Following screening and randomization at baseline, **rTMS** sessions were delivered every weekday for 3 weeks. **Consumption** - Self-report of the number of cigarettes per day (CPD). **Craving VAS:** Visual analog scale (VAS) before and after provocation (Pre- and Post-cue; VAS 1 and VAS2), and immediately after the rTMS session (Post-rTMS; VAS3). **Adverse Event:**  Daily adverse event monitoring.

**Daily Assessments:** Craving VAS, cigarettes per day, carbon monoxide (CO), Adverse Events,

**Weekly Assessments:** Fagerstrom test of nicotine dependence (FTND), The Minnesota nicotine withdrawal scale (MNWS-R), Urine cotinine & creatinine, MRI scan

1. **Statistical Power Consideration.** *Aim 1:* In selecting our target sample size, we balanced power considerations with the feasibility of execution since the UG3 is only two years long. To the best of our knowledge, we are not aware of any study that has compared the effect of mOFC-rTMS to the DLPFC-rTMS in smokers. *We do not expect a significant difference in Aim 1 at the UG3 phase. However, we hypothesize that at least one treatment results in a better clinical effect than the sham treatment by 5 cigarettes per day.* The primary efficacy of rTMS will be measured by the smoking quit rate and the number of cigarettes smoked per day at the end of treatment. Our preliminary, 10-session daily rTMS study showed that active rTMS reduces cigarette consumption when compared to sham rTMS (see Fig. 3 for details)([Li et al., 2020](#_ENREF_15)). The difference between the number of cigarettes smoked in the two groups of participants (daily 1Hz vs. 10-Hz rTMS) is 3 or more cigarettes per day (measured as a change from baseline at 21 days) the trial will move on to the next phase.  A sample size of 14 in each group will provide sufficient power for this trial, based on a futility rule for comparing the two groups, at 5% significance level, 80% power and assuming a standard deviation of 2.5 cigarettes per day (based on R21) change from baseline. The attrition rate for one previous 10 daily rTMS sessions study was 8% ([Amiaz et al., 2009](#_ENREF_1)). Our preliminary two-week rTMS study similarly showed an attrition rate of 9.5% (4/42)([Li et al., 2020](#_ENREF_15)). We anticipate the same attrition rate in this study, and therefore, we plan to achieve a total of 45 (15 in each group).  *Aim 2:* In this aim, our dependent measure is the difference of brain activity in the executive control and reward circuits between 1 Hz-rTMS over the left mOFC and 10 Hz rTMS over the left DLPFC. We computed the effect size by comparing the elevation in BOLD between the DLPFC TMS and the MPFC TMS (0.12 ± 0.03 and 0.07 ± 0.03 respectively) ([Hanlon et al., 2013](#_ENREF_11)). A total of 14 participants will ensure adequate power to detect differences in BOLD changes between two the sites with at least 90% power (2-sided type I error of 0.05). In another study, we computed the effect size by comparing in BOLD change between before and after one session rTMS (0.14 ± 0.27 and -0.38 ± 0.39 respectively). A total of 14 participants will ensure adequate power to detect differences in % BOLD changes (NAc) between pre and post rTMS with at least 90% power (2-sided type I error of 0.05). The attrition rate for our preliminary data was 9%. According to the attrition rate, we will enroll 15 participants to have sample power to detect a meaningful difference in BOLD signal. Since each participant will fulfill both aims, and because we are recruiting 45 participants for Aim 1, we will have more than sufficient power for Aim 2. For the exploratory Aim 3, since no data are available for power analysis, we will explore the relationship between the reductions in cigarette consumption and the normalized index of executive control and driving reward circuits in an exploratory fashion.
2. **Electric Sham rTMS (E-sham)**

**e Figure 2.** E**-**Sham_loop for the study.


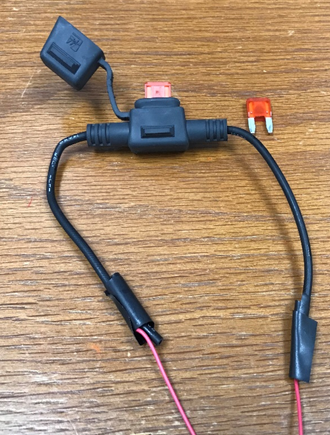


In one of our previous studies, we successfully developed an active sham system (E-sham) ([Arana et al., 2008](#_ENREF_2); [Borckardt et al., 2009](#_ENREF_3); [George et al., 2010](#_ENREF_10)). There is a built-in sham stimulation with the MagVenture TMS system; however, it wrongly delivers an electrical pulse to both active and sham patients. This results in more pain for those getting both active TMS and the active sham. We fixed this problem with this system by developing a double-blind loop to attach to the MagVenture double-blinded coil (e **Figure 2**). The participant and the treatment operator were both masked to the treatment group assignment in a double-blind manner, and all groups had similar pain sensations. To further assess the adequacy of the mask, TMS administrators, clinical raters, and patients completed "best guess" questionnaires, which assessed their best estimate of the treatment condition and their level of confidence in this estimate.

1. **Localizing the TMS coil over the left DLPFC or the left mOFC**

We reported that Resisting MRI ("resist the urge to smoke when you see the smoking-related pictures") could induce brain activity in the left prefrontal cortex ([Hartwell et al., 2011](#_ENREF_12)). Individual "resisting" scans were analyzed at an uncorrected p <0.05 within the mask of the left DLPFC (resist > crave). If no significantly activated voxel was found, we selected the MNI target (-42, 37, 31) that was reported in our previous study ([Li et al., 2020](#_ENREF_15)). We used a cue craving MRI scan ("allow yourself to crave when you see the smoking-related pictures") to identify the maximum voxel in mOFC for mOFC rTMS target. We used (-9, 53, 2) as a default targeted site if we did not find a significant activity in mOFC ([Li et al., 2024](#_ENREF_13)).

1. **E-field dosage method**.

Rather than titrating the stimulation intensity using a motor threshold, we used individual E-field modeling to determine optimal TMS intensity for each individual participant (SimNIBS 3.2) ([Opitz et al., 2011](#_ENREF_19); [Thielscher et al., 2011](#_ENREF_20); [Caulfield et al., 2021a](#_ENREF_6), [2021b](#_ENREF_7)). The personalized stimulation intensity was 85.0 V/m over the left DLPFC and 70.0 V/m over the mOFC based on prior modeling data in TUD at 120% motor threshold, as is typically used in standard clinical treatment. For safety considerations, we established a maximum stimulation intensity of 140% of the motor threshold.([Caulfield et al., 2021a](#_ENREF_6), [2021b](#_ENREF_7))

1. **Cue Provocation**

Previous studies have suggested that using provocation with real-life smoking cue exposure just prior to high-frequency stimulation can reduce nicotine dependence, as measured by the Fagerstrom Test for Nicotine Dependence ( FTND) ([Dinur-Klein et al., 2014](#_ENREF_8)). rTMS following smoking cue exposure may disrupt circuits associated with craving ([Li, Hartwell, Owens, et al., 2013](#_ENREF_16)). We performed a smoking cue exposure script just prior to the rTMS session and a smoking cue video during the rTMS session.

For the smoking cue exposure, we placed a pack of the individual's cigarettes and lighter in a tray and played a provocation cue recorded script. Before and after the script, we asked the participant to complete the nicotine craving Visual Analogue Scale (VAS). The script took 2 minutes. Craving was assessed using the VAS at three time points: 1) Before the provocation procedure, 2) After the provocation, and 3) After the rTMS session.

Smoking cue video: The in-house developed video was displayed during the 15 minutes of rTMS treatment. This video displayed actors performing cigarette-smoking-related behaviors (e.g., lighting up). The cues consisted of montages of videos, including cigarette-smoking-related behaviors (as cigarette advertisements). Music was being played during the video display.

*Pre-TMS Cue Provocation***:** We used structured 1.5 min exposure and interactions with real-life smoking paraphernalia (cigarettes, ashtray, lighter) ([Carpenter et al., 2014](#_ENREF_5)) immediately before each rTMS session. While rTMS was administered, subjects watched a 15-minute smoking cued video ([Li, Hartwell, Owens, et al., 2013](#_ENREF_16)) (scenes of individuals smoking in various environments) displayed on an iPad placed on a tripod at the foot of the treatment chair.

1. **Biomarkers**

We measured carbon monoxide (CO) levels before each TMS session using the Micro Smokerlyzer Breath Carbon Monoxide (CO) Monitor. Urine cotinine Levels were measured at baseline, before the 6th TMS session, before the 11th TMS session, before the 15^th^ TMS session, and at 1-month follow-up. Abstinence was defined as a self-report of no cigarettes smoked verified with urine cotinine levels < 200 ng/ml([Bramer & Kallungal, 2003](#_ENREF_4)) and CO < 5 parts per million (ppm)([Marrone et al., 2010](#_ENREF_18)). Abstinence was assessed at the end of the 3-week treatment and 1-month follow-up visit.

1. **Functional MRI**

*Smoking Cue Craving and Resisting Paradigm:* This well-validated paradigm, used by our team over the past 20 years, reliably elicits robust reward circuitry activation by cue craving ([Hartwell et al., 2011](#_ENREF_12); [Li, Hartwell, Borckardt, et al., 2013](#_ENREF_14); [Li et al., 2017](#_ENREF_17); [Li et al., 2024](#_ENREF_13)) as well as robust executive control circuitry activation by "resisting" craving ([Hartwell et al., 2011](#_ENREF_12)). Participants' urge to smoke cigarettes was rated by a hand pad (1-5) between blocks.

Each participant acquired a structural MPRAGE T1-weighted anatomical scan in 178 slices with a TR of 2250 ms, a TE of 4.18 ms, voxel dimensions of 1.0 x 1.0 x 1.0 mm, full-brain and cerebellar coverage, and no gap. After each volume was acquired, it was automatically exported in DICOM format from the MRI scanner computer to a separate computer for in-scan processing.([Hartwell et al., 2011](#_ENREF_12); [Li, Hartwell, Borckardt, et al., 2013](#_ENREF_14))

#### *Whole-Brain Analysis:* The d-oxygen-level-dependent (BOLD) functional MRI scans were converted into ANALYZE format using MRIcron and then further processed in MATLAB 9.3 (MathWorks, Sherborn, MA) with Statistical Parametric Mapping 12 (SPM12, Wellcome Department of Cognitive Neurology, London, UK). All volumes were realigned to the first volume. After realignment, for all subjects, movement across the 12-minute (328 time points) scan was less than 2 mm in 3 axes and less than 2 degrees in 3 orientations. The images were normalized stereotactically into a standard space with a resolution of 3 mm^3^ voxels using the averaged functional EPI image – the Montreal Neurological Institute (MNI) EPI template in SPM12. Subsequently, the data were smoothed with an anisotropic 8 mm^3^ Gaussian kernel and high-pass filtered (cut-off period=128s). At the first level of statistical analysis, using a boxcar function convolved with the modeled hemodynamic response function as the basic function for the general linear model ([Friston, 1994](#_ENREF_9)), we obtained contrast maps of the difference between smoking (either craving or resisting) and neutral cues for each subject. The six head movement parameters were included as confounds.

Subject-specific contrasts were entered into a second-level analysis to obtain a random effect analysis of activation effects across the entire group. We used a factorial design to do 2 (time: pre- vs. post-rTMS x 3 mixed model (stimulation condition: sham rTMS, active 10 Hz DLPFC rTMS, and active 1 Hz mOFC ). The cluster-level threshold was set at p < 0.05 using a family-wise error (FWE) rate correction for multiple comparisons, with a voxel-wise threshold of p < 0.001. Post hoc analyses: we performed a paired T-test with a covariable of reduction of CPD.

*Region of Interest Analysis and Extracting BOLD Signal:* To directly test the associated network-based hypotheses, we included region of interest (ROI)s for network analysis as follows. 1. Previous review papers reported the drive-reward and executive control connectivity in addiction research,([Volkow et al., 2011](#_ENREF_24); [Volkow et al., 2012](#_ENREF_23); [Volkow & Morales, 2015](#_ENREF_22)) , in which ROIs were derived from the Neurosynth platform (<http://neurosynth.org>) 2. Smoking cue exposure fMRI induced brain activity in the current study. 3. Resisting cue craving fMRI induced brain activity in the current study. 4. The left hemisphere (rTMS delivered side). A 6 mm spherical area was extracted within the ROIs, centered on the local maximum z value for each ROI. We measured Pearson's correlation coefficients (r) between the primary outcome and selected ROIs using the beta value from each ROI.

**RESULTS**

1. **Enrollment**

Of 234 potential participants who were contacted. 111 of them were for a phone screen, and 46 were eligible for the study. Of these 46 participants, 15 were assigned to receive Sham TMS (DLPFC or mOFC), 15 received active rTMS over the left DLPFC rTMS, and 16 received active rTMS over the mOFC. For analysis, completer cases were defined as randomized patients who received 15 sessions of rTMS treatment and underwent two MRI scans (baseline and post-rTMS). Overall, 35 study participants were included in the data analysis (n=9 sham, n=12 DLPFC, and n=14 mOFC).

1. **Objective Evaluation of Creatinine-Adjusted Cotinine**

**eFigure 3** The DLPFC Group has lower Urinary Cotinine Adjusted by Creatinine


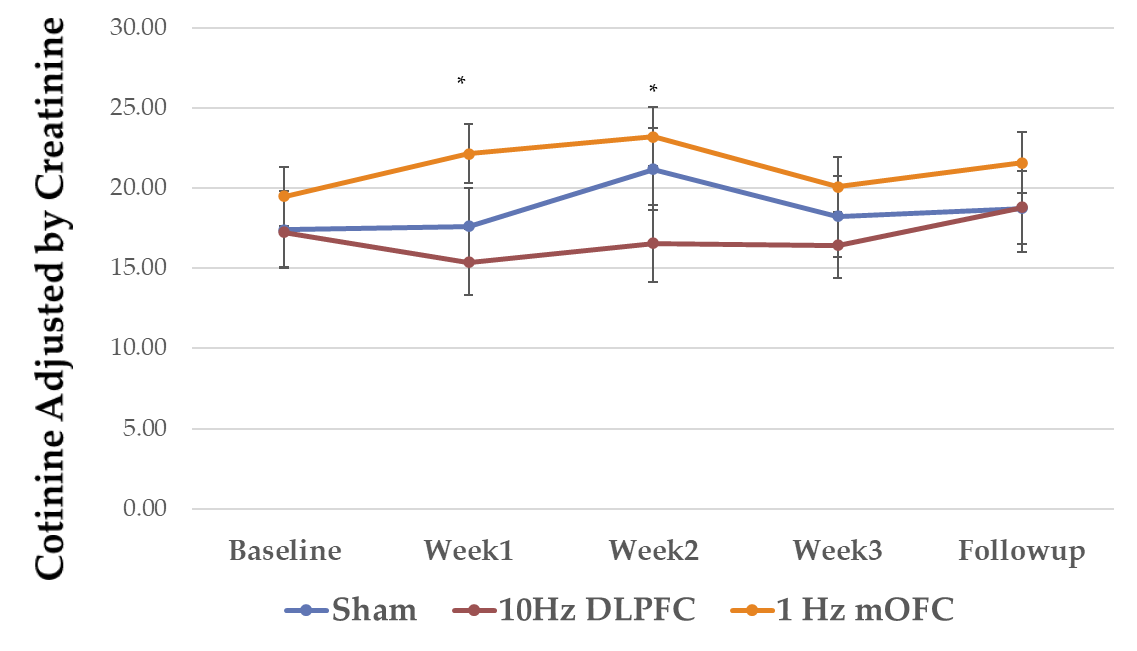


Urine cotinine and creatinine levels were measured at baseline, TMS6, TMS10, TMS15 and follow-up month 1. We used creatinine to adjust the cotinine([Thompson et al., 1990](#_ENREF_21)) with an equality formula- "Log(Urinary cotinine) = 0.407 x log(Urinary creatinine) + 0.586". The mixed model analysis revealed a significant treatment effect (sham:18.65 [1.12]; 10 Hz DLPFC:16.89[0.98]; 1 Hz mOFC:21.32[0.83]; F_(2,154)_ = 6.09, p = 0.003). Post-hoc analysis showed that the 10 Hz DLPFC group showed significantly less cotinine than the 1 Hz MOFC (p=0.001), especially in the first and the second week. (See e **Figure 2** for details. * p < 0.05)

1. **Smoking Abstinence by Treatment Group**

At the end of the 3-week rTMS treatment, 2 participants in 10 Hz DLPFC quit smoking during the treatment course. One sham participant and one 1 Hz mOFC treatment participant quit during the treatment course. One participant from 10 Hz DLPFC treatment quit smoking during the follow-up period. We found no significant treatment effect in smoking abstinence rate (X^2^ is 0.53, df =2, p = 0.76) and follow-up (X^2^ is 1.22, df =2, p = 0.54). (See e **Figure 3** for details)

**eFigure 4** Quit Rates in Three Treatment Groups
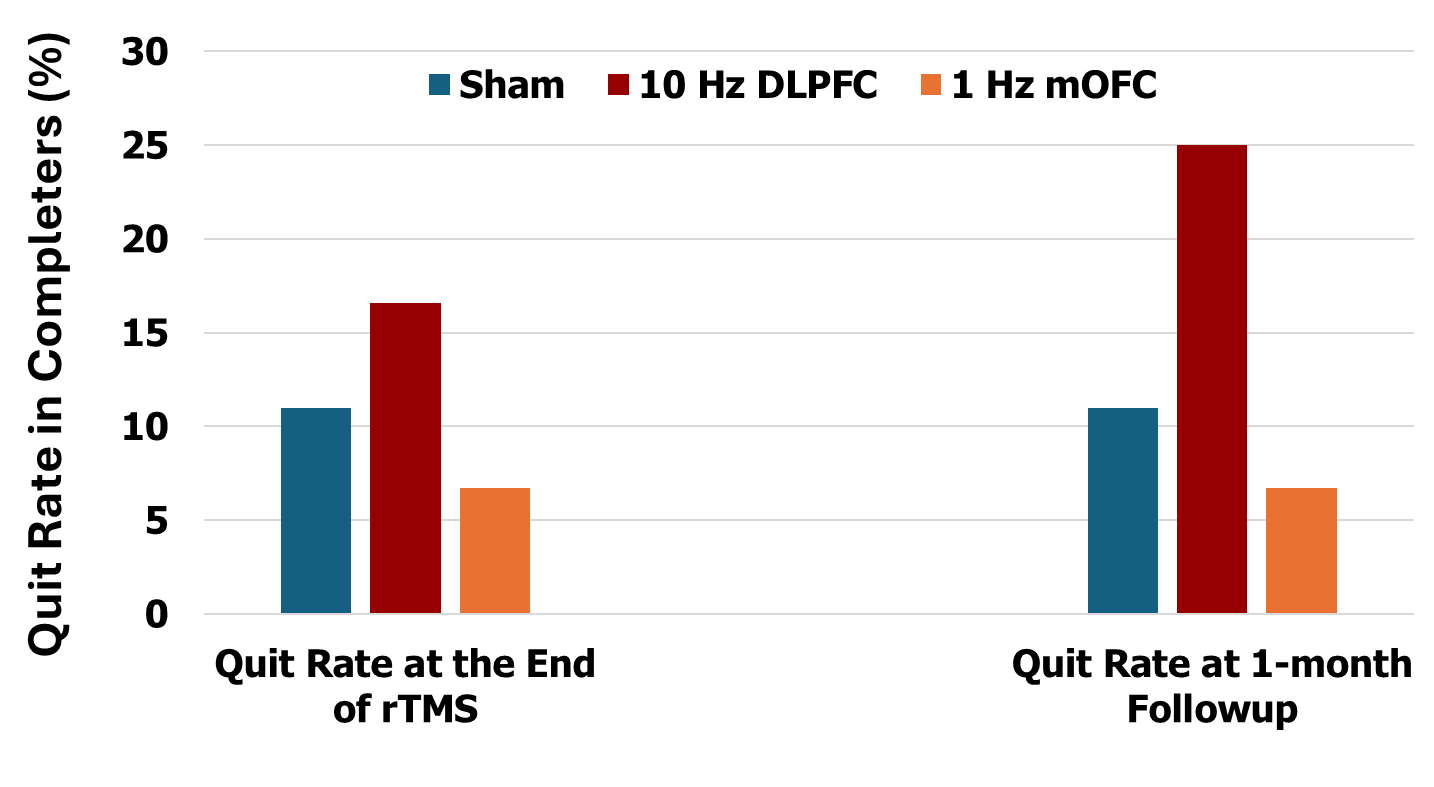


1. **Withdrawal symptoms (MNWS)**

Mixed model analysis revealed that no treatment effect was found between treatment groups in the reduction of MNWS (sham: 1.04[2.08], 10 Hz rTMS: 6.65[1.88], and 1 Hz rTMS: 5.21[1.71]; F_2,133_ = 2.12, P =0.12). No difference between visiting time was found in the reduction of MNWS (F_3,133_ = 2.12, P =0.42).

1. **Nicotine dependence (FTND)**

Using the reduction of FTND score, a mixed model analysis revealed that the main effect of treatment time, which decreased during the treatment course (0.001[0.34], 1.43[0.35], 1.65[0.34],1.86[0.35], and 2.15[0.36]; F_4,158_ = 5.88, P =0.0002). No main effect of treatment groups was found in the reduction of FTND (F_2,158_ = 5.88, P =0.097).

**eTable 1.** Adverse Events Report
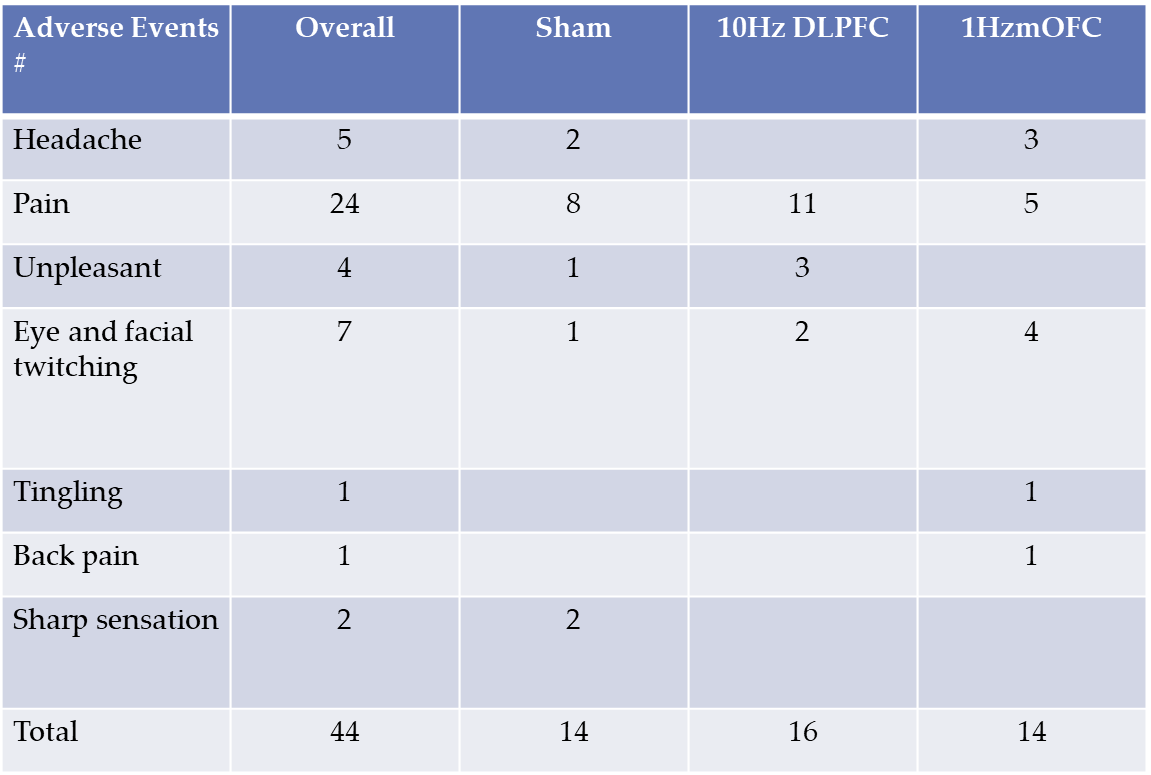


1. **Treatment Adherence, Visit Attendance, And Adverse Events**

Regarding the active sham control study, 17 out of 35 participants reported adverse events (AE). 17 participants with AE included 6 (37%) from sham, 5 (26%) from DLPFC), and 6 (37%) from mOFC. There were no differences between treatment groups (X^2^ =0.025, p=0.98). The number of AEs is reported in e **Table 1**. Headache (5), discomfort or unpleasant feelings (4), pain (24), and eye and facial twitching (7) were the most popular side effects. No participant needed treatment for the side effects. Pain or unpleasant feelings were solved by decreasing intensity.

1. **Whole Brain Analysis Results**

Pre-TMS treatment, we found that cue craving scan induced brain activity in the mOFC at p < 0.05 using family-wise error (FWE) rate correction for multiple comparisons, with voxel-wise threshold p < 0.001.

**Cue Craving Scans:** An important quality step is whether we were able to activate the craving network with our cue-induced craving. **Figure 5 and e Table 2** show the difference between when subjects saw cigarette cues and when subjects saw neutral cues. They were instructed to crave. (Figure 8 is from the baseline (n=35, all subjects): The red regions are more active with craving. The blue regions are decreased during craving. All contrast maps at FWE for cluster and 0.001 for voxel.) The results were consistent with our previous findings ([Hartwell et al., 2011](#_ENREF_12); [Li, Hartwell, Borckardt, et al., 2013](#_ENREF_14)).

**eFigure 6** Our resisting craving paradigm caused brain activation in the insula, MPFC and DLPFC. These are the regions the subjects used to resisting craving. (All subjects at baseline, neutral minus smoke) (n = 35)


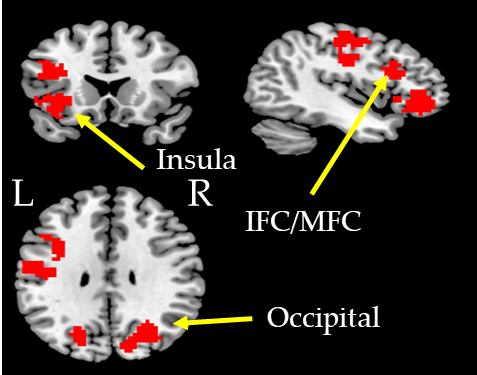


**eFigure** 5 Our activation paradigm caused brain activation in the craving network. (All subjects, baseline, craving minus neutral). (n = 35)


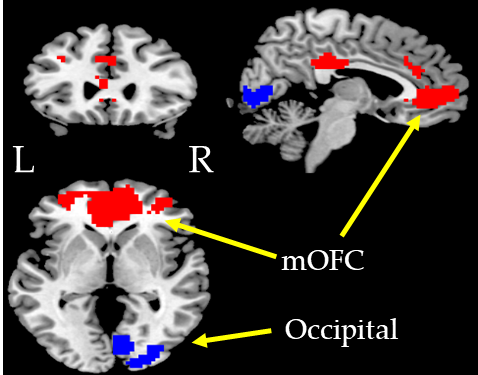


**Resisting to Cue Scans:** It was important to show that cigarette cravings using our cues activated the addiction network. But perhaps more importantly, quitting cigarettes may be associated with resisting the urge to smoke. For this analysis of target engagement, we had subjects resist craving in the scanner while seeing the same cigarette cues.

**Figure 6 and Table 2** present more active areas when subjects resisted smoking cues less than neutral ones (FWE corrected for cluster and 0.001 for voxel threshold). It means that resisting to craving cues induces brain activity in the left insula, the inferior and middle prefrontal cortex.

Analyzing the resisting scan data, we found that Resisting cue craving induced brain activity in the left prefrontal cortex as well as insula. (0.05 FWE for cluster and voxel threshold p<0.001. Note: no activity was found at the voxel threshold p<0.001)).

**e Table 2**. Compared to neutral, smoking cue induced brain activity in smokers at baseline Cue Craving and Resisting Craving. Compared to neutral, smoking cue induced brain activity at before treatment and after treatment in three rTMS groups (sham, 10 Hz DLPFC, and 1 Hz mOFC) (voxel threshold = uncorrected .001, cluster corrected with family-wise error .05)

X, Y, and Z refer to the coordinates of peak t statistic for each cluster in the Montreal Neurological Institute Space.

| **Whole Brain Analysis for 35 Completed Participants at Baseline** | | | | | | |
| --- | --- | --- | --- | --- | --- | --- |
| x | y | z | T value | Cluster size | Uncorrected P | Brain region |
| *Baseline Cue Craving Scan for all 35* | | | | | | |
| Smoke greater than Neutral (0.05FWE for cluster and voxel 0.001) | | | | | | |
| -9 | 47 | -1 | 5.84 | 1236 | 0.000 | L medial orbitofrontal cortex/ACC |
| 6 | 64 | -7 | 5.73 |  |  | R medialorbitofronatl cortex |
| -9 | 38 | 11 | 5.68 |  |  | L anterior cingulate cortex |
| Smoke less than Neutral (0.05FWE for cluster and voxel 0.001) | | | | | | |
| 33 | -76 | 23 | 4.16 | 107 | 0.000 | R occipital_middle |
| *Baseline Resisting Cue Craving Scan for all 35* | | | | | | |
| Smoke greater than Neutral (0.05FWE for cluster and voxel 0.001) No activity. | | | | | | |
| Smoke less than Neutral (50 voxels for cluster and voxel 0.001) | | | | | | |
| -45 | 38 | 2 | 4.20 | 63 | 0.000 | L Frontal_inf_tri |
| -57 | -7 | 38 | 3.99 | 52 | 0.000 | L Postcentral |
|  |  |  |  |  |  |  |
| **Whole Brain Analysis for 12 Completed Participants Pre and Post 10 Hz DLPFC** | | | | | | |
| *Decreased Brain Activity for Craving Scan after 10 Hz DLPFC* | | | | | | |
| -18 | 42 | -12 | 8.80 | 144 | 0.000 | L Frontal_Sup_Orb |
| *Increased Brain Activity for Craving Scan after 10 Hz DLPFC* | | | | | | |
| -15 | -20 | 42 | 7.84 | 1452 | 0.000 | LCingulum_Middle |
| -33 | 21 | 33 | 6.66 |  |  | L Frontal_Middle(DLPFC) |
| -30 | -12 | 42 | 6.61 |  |  | L Precentral Gyrus |
|  | | | | | | |
| **Whole Brain Analysis for 9 Completed Participants Pre and Post Sham** | | | | | | |
| No activity (0.05FWE for cluster and voxel 0.001) in craving MRI | | | | | | |
| No activity (0.05FWE for cluster and voxel 0.001) in resisting MRI | | | | | | |
| **Whole Brain Analysis for 14 Completed Participants Pre and Post 1 Hz mOFC (No Significant )** | | | | | | |
| No activity (0.05FWE for cluster and voxel 0.001) in craving MRI | | | | | | |
| No activity (0.05FWE for cluster and voxel 0.001) in resisting MRI | | | | | | |
| **Factor Design for Craving MRI (TMS conditions: 10 Hz, 1 Hz, and sham; treatment time: pre and post)** | | | | | | |
| No interaction activity (0.05FWE for cluster and voxel 0.001) in craving MRI | | | | | | |
| **Factor Design for Resisting MRI (TMS conditions: 10 Hz, 1 Hz, and sham; treatment time: pre and post)** | | | | | | |
| No interaction activity (0.05FWE for cluster and voxel 0.001) in resisting MRI | | | | | | |

1. **Blinding**

The subject and the treatment operator were masked to the treatment group assignment. Research staff who were not involved in the TMS administration, data acquisition, or data analysis prepared the patient for treatment (active or sham) according to the randomization chart generated by a statistician. This arrangement allowed the principal investigator, TMS administrator, and clinical rater to remain blind to rTMS treatments. To assess the adequacy of the mask, at the end of treatment, TMS administrators and patients completed items querying "best guess" as to the treatment condition and their level of confidence in this guess.

At the end of the first rTMS session visit, we asked participants to guess the treatment condition. Participants were asked the following questions. "(1) Strong belief I received Active Treatment; (2) Moderate belief I received Active Treatment; (3) Don't know; (4) Moderate belief I received Sham Treatment; (5) Strong belief I received Sham Treatment. 16 out of 35 participants selected "I don't know". 14 participants selected "Strong belief I received Active Treatment". 3 Participants selected "Moderate belief I received Active Treatment". One selected "Moderate belief I received Sham Treatment". One selected "Strong belief I received Sham Treatment."

**e Table 3. The integrity of the blind**

| End Point | Patient  Sham Active 10 Hz Active 1 Hz | P value | Treater  Sham Active 10Hz Active 1 Hz | | P value |  |
| --- | --- | --- | --- | --- | --- | --- |
|  | | | | |  | |
| Patients, No.  End of treatment accurate guess (%)  Correct guess when extremely confident, No  Confidence in guess, No. (%)  Extremely  Considerably  Moderately  Slightly  Not at all | 9 12 14  3(33) 10(83) 11(79)  0 4 3  1 (11) 7(58) 6(43)  2(22) 0(0) 2(14)  3(33) 4(33) 5(36)  0(0) 0(0) 1(7)  3(33) 1(8) 0 | .03  .09  .99  . | 9  5(56)  0  0 (0)  0(0)  3(33)  3(33)  3(33) | 12 14  11(92) 11(79)  1(8) 1(7)    1(8) 1(7)  1(8) 2(14)  3(35) 7(50)  3(40) 1(7)  4(15) 3(21) | .15  .48  .84  .74 |  |
|  | | | | |  | |

This was a well-blinded study at the treatment start. By the end of the study, participants correctly guessed the sham condition less than either of the active treatment groups (p =0.03). Approximately 80% of participants in both the 10 Hz DLPFC and 1 Hz mOFC groups correctly identified their treatment, which was notably higher than in the sham group. However, there is no significant difference between the two active groups. Confidence ratings did not differ significantly between treatment groups. Given the study design- with one sham group (expected correct guess rate of 33%) and two active groups (expected correct guess rate of 66%)- we believe the blinding was successful overall (p = 0.89). Additionally, the operator's accuracy was not significantly different across groups.

References

Amiaz, R., Levy, D., Vainiger, D., Grunhaus, L., & Zangen, A. (2009). Repeated high-frequency transcranial magnetic stimulation over the dorsolateral prefrontal cortex reduces cigarette craving and consumption. *Addiction, 104*(4), 653-660. doi: 10.1111/j.1360-0443.2008.02448.x

Arana, A. B., Borckardt, J. J., Ricci, R., Anderson, B., Li, X., Linder, K. J., et al. (2008). Focal electrical stimulation as a sham control for repetitive transcranial magnetic stimulation: Does it truly mimic the cutaneous sensation and pain of active prefrontal repetitive transcranial magnetic stimulation? *Brain Stimul, 1*(1), 44-51. doi: 10.1016/j.brs.2007.08.006

Borckardt, J. J., Linder, K. J., Ricci, R., Li, X., Anderson, B., Arana, A., et al. (2009). Focal electrically administered therapy: device parameter effects on stimulus perception in humans. *J ECT, 25*(2), 91-98. doi: 10.1097/YCT.0b013e318183c6a4

Bramer, S. L., & Kallungal, B. A. (2003). Clinical considerations in study designs that use cotinine as a biomarker. *Biomarkers, 8*(3-4), 187-203. doi: 10.1080/13547500310012545

Carpenter, M. J., Saladin, M. E., Larowe, S. D., McClure, E. A., Simonian, S., Upadhyaya, H. P., et al. (2014). Craving, cue reactivity, and stimulus control among early-stage young smokers: effects of smoking intensity and gender. *Nicotine Tob Res, 16*(2), 208-215. doi: 10.1093/ntr/ntt147

Caulfield, K. A., Li, X., & George, M. S. (2021a). Four electric field modeling methods of Dosing Prefrontal Transcranial Magnetic Stimulation (TMS): Introducing APEX MT dosimetry. *Brain Stimul, 14*(4), 1032-1034. doi: 10.1016/j.brs.2021.06.012

Caulfield, K. A., Li, X., & George, M. S. (2021b). A reexamination of motor and prefrontal TMS in tobacco use disorder: Time for personalized dosing based on electric field modeling? *Clin Neurophysiol, 132*(9), 2199-2207. doi: 10.1016/j.clinph.2021.06.015

Dinur-Klein, L., Dannon, P., Hadar, A., Rosenberg, O., Roth, Y., Kotler, M., et al. (2014). Smoking cessation induced by deep repetitive transcranial magnetic stimulation of the prefrontal and insular cortices: a prospective, randomized controlled trial. *Biol Psychiatry, 76*(9), 742-749. doi: 10.1016/j.biopsych.2014.05.020

Friston, K. J. H., A.P; Worsley, K.J; Poline, J.P.; Frith, R.S.J. (1994). Statistical parametric maps in functional imaging: a general linear approach. *Hum Brain Mapp, 2*(4), 189-210.

George, M. S., Lisanby, S. H., Avery, D., McDonald, W. M., Durkalski, V., Pavlicova, M., et al. (2010). Daily left prefrontal transcranial magnetic stimulation therapy for major depressive disorder: a sham-controlled randomized trial. *Arch Gen Psychiatry, 67*(5), 507-516. doi: 10.1001/archgenpsychiatry.2010.46

Hanlon, C. A., Canterberry, M., Taylor, J. J., DeVries, W., Li, X., Brown, T. R., et al. (2013). Probing the frontostriatal loops involved in executive and limbic processing via interleaved TMS and functional MRI at two prefrontal locations: a pilot study. *PLoS One, 8*(7), e67917. doi: 10.1371/journal.pone.0067917

Hartwell, K. J., Johnson, K. A., Li, X., Myrick, H., LeMatty, T., George, M. S., et al. (2011). Neural correlates of craving and resisting craving for tobacco in nicotine dependent smokers. *Addict Biol, 16*(4), 654-666. doi: 10.1111/j.1369-1600.2011.00340.x

Li, X., Caulfield, K. A., Hartwell, K. J., Henderson, S., Brady, K. T., & George, M. S. (2024). Reduced executive and reward connectivity is associated with smoking cessation response to repetitive transcranial magnetic stimulation: A double-blind, randomized, sham-controlled trial. *Brain Imaging Behav, 18*(1), 207-219. doi: 10.1007/s11682-023-00820-3

Li, X., Hartwell, K. J., Borckardt, J., Prisciandaro, J. J., Saladin, M. E., Morgan, P. S., et al. (2013). Volitional reduction of anterior cingulate cortex activity produces decreased cue craving in smoking cessation: a preliminary real-time fMRI study. *Addict Biol, 18*(4), 739-748. doi: 10.1111/j.1369-1600.2012.00449.x

Li, X., Hartwell, K. J., Henderson, S., Badran, B. W., Brady, K. T., & George, M. S. (2020). Two weeks of image-guided left dorsolateral prefrontal cortex repetitive transcranial magnetic stimulation improves smoking cessation: A double-blind, sham-controlled, randomized clinical trial. *Brain Stimul, 13*(5), 1271-1279. doi: 10.1016/j.brs.2020.06.007

Li, X., Hartwell, K. J., Owens, M., Lematty, T., Borckardt, J. J., Hanlon, C. A., et al. (2013). Repetitive transcranial magnetic stimulation of the dorsolateral prefrontal cortex reduces nicotine cue craving. *Biol Psychiatry, 73*(8), 714-720. doi: 10.1016/j.biopsych.2013.01.003

Li, X., Sahlem, G. L., Badran, B. W., McTeague, L. M., Hanlon, C. A., Hartwell, K. J., et al. (2017). Transcranial magnetic stimulation of the dorsal lateral prefrontal cortex inhibits medial orbitofrontal activity in smokers. *Am J Addict, 26*(8), 788-794. doi: 10.1111/ajad.12621

Marrone, G. F., Paulpillai, M., Evans, R. J., Singleton, E. G., & Heishman, S. J. (2010). Breath carbon monoxide and semiquantitative saliva cotinine as biomarkers for smoking. *Hum Psychopharmacol, 25*(1), 80-83. doi: 10.1002/hup.1078

Opitz, A., Windhoff, M., Heidemann, R. M., Turner, R., & Thielscher, A. (2011). How the brain tissue shapes the electric field induced by transcranial magnetic stimulation. *Neuroimage, 58*(3), 849-859. doi: 10.1016/j.neuroimage.2011.06.069

Thielscher, A., Opitz, A., & Windhoff, M. (2011). Impact of the gyral geometry on the electric field induced by transcranial magnetic stimulation. *Neuroimage, 54*(1), 234-243. doi: 10.1016/j.neuroimage.2010.07.061

Thompson, S. G., Barlow, R. D., Wald, N. J., & Van Vunakis, H. (1990). How should urinary cotinine concentrations be adjusted for urinary creatinine concentration? *Clin Chim Acta, 187*(3), 289-295. doi: 10.1016/0009-8981(90)90114-8

Volkow, N. D., & Morales, M. (2015). The Brain on Drugs: From Reward to Addiction. *Cell, 162*(4), 712-725. doi: 10.1016/j.cell.2015.07.046

Volkow, N. D., Wang, G. J., Fowler, J. S., & Tomasi, D. (2012). Addiction circuitry in the human brain. *Annu Rev Pharmacol Toxicol, 52*, 321-336. doi: 10.1146/annurev-pharmtox-010611-134625

Volkow, N. D., Wang, G. J., Fowler, J. S., Tomasi, D., & Telang, F. (2011). Addiction: beyond dopamine reward circuitry. *Proc Natl Acad Sci U S A, 108*(37), 15037-15042. doi: 10.1073/pnas.1010654108
